# Potassium ferrate’s disinfecting ability: a study on human adenovirus, *Giardia duodenalis,* and microbial indicators under varying pH and water temperature conditions

**DOI:** 10.2166/wh.2024.087

**Published:** 2024-05-21

**Authors:** Laura A. Boczek, Michael W. Ware, Mark R. Rodgers, Hodon Ryu

**Affiliations:** United States Environmental Protection Agency, Office of Research and Development, 26 W. Martin Luther King Dr, Cincinnati, OH 45268, USA

**Keywords:** microbial inactivation, potassium ferrate, waterborne pathogens

## Abstract

Ferrate (Fe(VI): ${\mathrm{HFeO}}_{4}^{-}∕{\mathrm{FeO}}_{4}^{2-}$), a potent oxidant, has been investigated as an alternative chemical disinfectant in water treatment due to its reduced production of disinfection by-products. In this study, we assessed the disinfecting ability of potassium ferrate against a variety of microorganisms, including waterborne pathogens, under varying pH and water temperature conditions. We presented CT values, a metric of ferrate concentrations (C) and contact time (T), to quantify microbial inactivation rates. Among the tested microorganisms, human adenovirus was the least resistant to ferrate, followed by waterborne bacteria such as *Escherichia coli* and *Vibrio cholerae,* and finally, the protozoan parasite *Giardia duodenalis.* We further investigated the impact of two pH values (7 and 8) and two temperatures (5 and 25 °C) on microbial inactivation rates, observing that inactivation rates increased with lower pH and higher temperature. In addition to showcasing ferrate’s capacity to effectively inactivate a range of the tested microorganisms, we offer a ferrate CT table to facilitate the comparison of the effectiveness of various disinfection methods.

## 1. INTRODUCTION

Drinking water treatment plants employ numerous methods to remove chemical and microbial contaminants and provide safe water to the consumer. One of many processes along the treatment train includes disinfection, which inactivates waterborne pathogens. In the United States, the most common disinfectants used in drinking water treatment are halogenated chemicals such as chlorine and chloramines. These chemicals are very effective at treating a wide variety of microorganisms, provide a disinfectant residual in the distribution system, and are relatively inexpensive. However, they produce disinfection by-products (DBPs), some of which are reasonably anticipated to be carcinogenic in humans (U.S. EPA 2006; Sedlak & von Gunten 2011). DBP formation continues to be a problem in drinking water treatment, and as such alternative methods for disinfection are always being sought.

Ferrate(Fe(VI): ${\text{HFeO}}_{4}^{-}∕{\text{FeO}}_{4}^{2-}$) has been investigated as a possible alternative chemical disinfectant in drinking and wastewater applications (Sharma 2002; Lee & von Gunten 2010). It is a strong oxidant (Sharma & Bielski 1991; Sharma 2007) with relatively low reactivity with organic matter and bromide ions, which consequently leads to reduced production of DBPs (DeLuca et al. 1983; Schuck et al. 2006; Sharma 2007, 2010; Lee et al. 2008; Lee & von Gunten 2010; Jiang et al. 2016). Ferrate has a relatively high redox potential (e.g., +2.2 for the ${\text{HFeO}}_{4}^{2-}$ species) compared to other oxidants such as ozone (+2.08) and chlorine (+1.39) (Jiang 2007). Moreover, the by-product formed is a non-toxic ferric ion (e.g., insoluble ferric oxyhydroxide), which can enhance the physical removal of microorganisms and DBP precursors by coagulation in tandem with oxidation/disinfection (Jiang & Lloyd 2002; Jiang et al. 2016).

A vast majority of ferrate disinfection studies have focused on degrading or oxidizing chemical contaminants in water treatment (Graham et al. 2004; Lee et al. 2005a, 2009; Sharma et al. 2006, 2012, 2013; Li et al. 2008; Sharma et al. 2008; Osathaphan et al. 2011; Yang et al. 2012; Anquandah et al. 2013). Some studies have looked at microbial inactivation but mainly on microbial indicators such as fecal coliforms (Sharma et al. 2005), *Escherichia coli* (Waite 1979; Jiang & Wang 2003; Cho et al. 2006), endospores (Franklin 1998; Makky et al. 2011), and bacteriophages (Schink & Waite 1980; Kazama 1995, 1994; Hu et al. 2012). These studies did not directly target waterborne pathogens and used contact time (*T*) as a dependent variable instead of ferrate concentrations and CT values. CT values are commonly used in disinfection studies and by regulators to develop disinfectant standards. Without CT values, it is hard to compare the effectiveness of different disinfection methods. Ferrate auto-decomposes in water, especially at lower pHs but also at pH values in the range of 7–8 that are relevant to water treatment. The potential for a considerable and rapid decrease in concentration necessitates that ferrate disinfection be assessed on a CT basis and not simply dose and reaction time.

Despite the multi-functional properties of ferrate that make it an attractive option for water disinfection, particularly as an alternative to chlorine, its use has been limited due to cost and stability concerns. However, new potassium ferrate products (>90% pure potassium ferrate salt) have recently entered the market. This product is a highly pure, stable solid that is much less expensive than other potassium ferrate products, providing practical benefits that address the aforementioned limitations and sparking renewed interest in its use for water disinfection. In this study, we assessed the disinfecting ability of potassium ferrate against a range of microorganisms, including waterborne pathogens, under varying pH and water temperature conditions and provided CT values for inactivating the tested microorganisms. To our knowledge, this is the first report to demonstrate the efficacy of ferrate in inactivating waterborne pathogens such as human adenovirus and *Giardia duodenalis* cysts.

## 2. MATERIALS AND METHODS

### 2.1. Ferrate chemistry

#### 2.1.1. Preparation of potassium ferrate stock solution

The ferrate stock solution with a nominal initial concentration of approximately 1 g/L (as ${\text{FeO}}_{4}^{2-}$) was prepared by adding 0.174 g of potassium ferrate (GFS Chemicals, Powell, OH, USA) into 100 mL of pH 9.0 chlorine demand-free (CDF) buffer. A desired working concentration of potassium ferrate in each reaction beaker is 5 mg$-{\text{FeO}}_{4}^{2^{-}}∕L$, which corresponds to 2.3 mg-Fe/L.

#### 2.1.2. Ferrate measurements

Ferrate was measured using 2,2′-azino-bis (3-ethylbenzothiazoline-6-sulphonic acid) or ABTS. Absorbance at 415 nm for ABTS is the preferred method of measurement (Lee et al. 2005b). The method consists of adding a Fe(VI)-containing sample to a mixture of ABTS and acetate buffer. Oxidation of the ABTS by Fe(VI) causes the formation of an ABTS radical, which is dark green and absorbs visible light at several prominent wavelengths including 415 nm. At proper time intervals, a 3.8 mL aliquot of the test beaker was removed and placed into a 15 mL conical centrifuge tube that contained 1 mL of an acetate buffer with 200 μL of ABTS reagent (added immediately before 3.8 mL of the test beaker solution). The colorized ABTS solution is then transferred to a quartz cuvette and measured in a UV–VIS or VIS spectrophotometer at 415 nm.

#### 2.2. Propagation and enumeration of testing microorganisms

The selected microorganisms included waterborne bacteria (three strains of *E. coli* including a pathogenic O157 strain and three strains of *Vibrio cholerae*), protozoan parasite (*G. duodenalis* cysts), and enteric virus (human adenovirus type 2 (HAdV2)). Membrane filtration, animal infectivity using Mongolian Gerbils, and tissue culture infectivity assays were used for the detection of the respective test microorganisms.

#### 2.2.1. Bacteria

Three strains of *E. coli* (ATCC 25922, ATCC 35323, and ATCC 35150 (O157)) and one strain of *V. cholerae* (ATCC 14033) were purchased from American Type Culture Collection (ATCC, Manassas, VA, USA). Additionally, two environmental strains of *V. cholerae* such as 6706-Smooth and 2614-LA were obtained from our current culture collection held at the EPA AWBERC facility in Cincinnati, OH. The rehydrated cultures with 5 mL of brain heart infusion broth (BHI broth) were inoculated onto brain heart infusion agar (BHIA) plates, and the plates were incubated at 35 °C for 18–24 h. Single colonies were transferred to 6 mL of BHI broth and then incubated overnight at 35 °C. Bacteria samples were assayed using the spread plate method (APHA 2005). The plates were incubated for 18–24 h at 35 °C. The number of colony-forming units present was counted.

#### 2.2.2. Adenovirus

HAdV2 was obtained from the American Type Tissue Collection (ATCC VR-846, Manassas, VA) and propagated in A549 human lung carcinoma cells (ATCC CCL-185). Briefly, A549 cells were infected with stock HAdV2, and the cells were incubated for up to 2 weeks or until cytopathogenic effects were observed. Cell lysates were centrifuged at 2,500 × g for 30 min to remove cell debris and then the supernatant containing virus was centrifuged at 10,000 × g for 10 min to remove any remaining small cellular debris. In order to remove any potential viral clumps, remove any remnant of growth media, and further purify the viral stock, the virus supernatant was further concentrated using celite, as described by McMinn et al. (2012). Following celite concentration, viral stocks were further concentrated using 30 kDa MWCO Vivaspin-20 ultrafilters (Sartorius-Stedim, Aubagne, France), as described previously (Cashdollar et al. 2013). Viral stocks were stored at −80 °C until ferrate disinfection experiments.

HAdV2 was enumerated using the Integrated Cell Culture Quantitative PCR (ICC-qPCR), as described in Ryu et al. (2015), which quantifies infectious adenoviruses from viral DNA harvested from a cell culture monolayer. Viral DNA from cell harvest with infectious viruses was extracted and purified with the DNeasy 96 Blood and Tissue Kit (QIAGEN, Valencia, CA, USA) according to the manufacturer’s instructions. DNA extracts were stored at −20 °C until the qPCR assay. The qPCR assay was performed in 25-μL reaction mixtures containing 1× TaqMan universal PCR master mix with AmpErase uracil-N-glycosylase using a 7900 HT Fast Real-Time Sequence Detector (Applied Biosystems, Foster City, CA, USA).

The ICC-qPCR standard curve was constructed using five 10-fold serial dilutions from the HAdV2 stock as described above. Briefly, based on the quantities resulting from the ICC-qPCR assay (x) and the original number of infectious HAdV2 spiked into each dilution (y), the regression analysis was performed to determine unknown parameter (a) in the standard curve (i.e., y=ax+b). The concentrations of infectious HAdV2 in pre-inoculation samples (i.e., the dependent variable, y = the original virus concentration prior to replication) were estimated using the regression equation with ICC-qPCR quantities (i.e., the independent variable, x). The estimated y values were used to calculate the log inactivation of HAdV2.

#### 2.2.3. *Giardia* cysts

*G. duodenalis* (H3 strain; Assemblage B) cysts were propagated in immunocompromised CF-1 mice (Ware & Villegas 2019). Cysts were purified by sucrose flotation followed by Percoll (GE Healthcare Life Sciences, Marlborough, MA, USA) sedimentation (Belosevic et al. 1983; Sauch 1984) and were used within 7 days of collection. Disinfection studies were performed in age-matched female Mongolian gerbils (35–40 g) from Charles River Laboratories (Wilmington, MA, USA). Gerbils were caged individually to prevent cross-contamination between animals since *G. duodenalis* is transmitted fecal-orally. The infectivity most probable number (MPN)/10^6^ cysts of control and disinfected cysts were determined by the infection response of animal cohorts to varying cyst doses by oral gavage (Shin et al. 2010). The preparation of cyst doses by flow cytometry and monitoring of animal infection (cysts or trophozoites) was previously described in Hayes et al. (2003), except that the FACS Aria II (Becton Dickinson, Franklin Lakes, NJ, USA) flow cytometer was used. All animal studies were approved and overseen by the Cincinnati U.S. EPA Institutional Animal Care and Use Committee.

### 2.3. Ferrate disinfection experiments

Bench-scale disinfection studies measuring the CT of the disinfectant and the log inactivation of microorganisms were conducted by using stock solutions of potassium ferrate as the ferrate source. Experiments were conducted using sterile CDF buffer at pH values of 7 and 8 and at 5 and 25 °C. Inactivation experiments are initiated by adding an aliquot of the ferrate stock solution to solutions of the bacterial culture buffered to the desired pH. For example, 200 mL of CDF buffer pH 7.0 or 8.0 is added to each 600 mL sterile beaker with stir bars. The beakers containing the testing microorganisms are constantly stirred using a magnetic stir plate. The reaction is held at a constant temperature either on the bench top for 25 °C or in a water bath for 5 °C. One mL of the washed bacterial, viral, or *Giardia* cultures is added to each beaker and allowed to stir and disperse for at least 1 min. An aliquot of the ferrate stock solution is then added to the microbial reactors under constant stirring conditions.

As the ferrate stock solution continually decays, the actual stock concentration for inactivation experiments is not accurately known. Accordingly, the initial ferrate concentration in the microbial reactions should be determined for any experiments by performing a control experiment, which was performed immediately prior to dosing of the microbial reactions. This control consists of an aliquot of the ferrate stock solution that is added to pH 9 CDF. Under pH 9 conditions and with the typical Fe(VI) concentrations used, the Fe(VI) remains stable and negligible decay of Fe(VI) is expected between the time of preparation and ABTS sampling. Hence, this control sample constitutes a good estimate of the initial ferrate concentration of the microbial reactions.

The disinfectant is allowed to react with the testing microorganisms for five different time intervals including time 0. The four time intervals are needed to achieve enough information to determine the inactivation rates of the testing microorganisms caused by the exposure to ferrate. At the end of each time frame, the ferrate reaction is neutralized with 0.5 mL of sodium thiosulfate (10% w/v).

### 2.4. Data presentation and inactivation kinetics

Triplicate tests were performed with each microbial stock of the testing microorganisms, and their ${\text{Log}}_{10}$ inactivations (I) were defined by the following equation:

$$I=-\log_{10}\left(\frac{N_{d}}{N_{0}}\right)$$

where $N_{0}$ and $N_{d}$ are the initial concentration and the concentration of culturable/infectious microorganisms with disinfection treatment, respectively. Values for inactivation kinetics were calculated using multiple CTs (i.e., mg/L as Fe × min) as described by the Chick-Watson model. The data points were fitted by regression analysis, and inactivation rate constants (k) [L/(mg-Fe × min)] were estimated according to the following equation:

$$\left(\frac{N_{d}}{N_{0}}\right)=e^{-k\text{CT}}$$

## 3. RESULTS AND DISCUSSION

**Figure 1 ∣:**
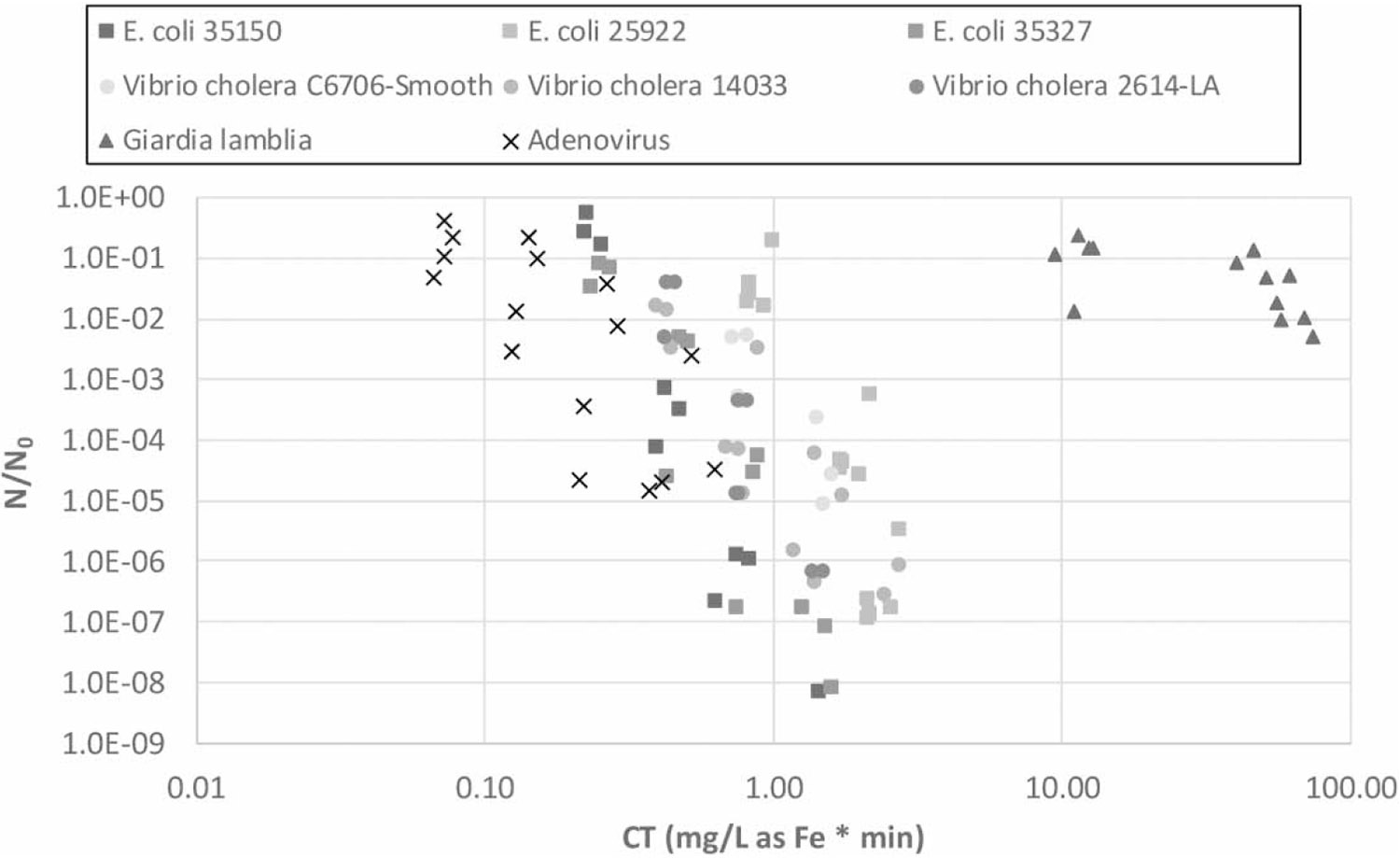
${\mathrm{Log}}_{10}$ inactivation of the testing microorganisms by ferrate disinfection under optimum performance conditions (i.e., at pH = 7 and temperature = 25 °C).

Ferrate disinfection experiments were performed against selected testing microorganisms under different water pH and temperature conditions. The inactivation rate constants (k) and CT values are presented in Tables 1 and 2. The k values ranged from 0.064 to 36.2 [L/(mg-Fe × min)], with lower values indicating higher resistance to ferrate. For instance, the protozoan parasite *G. duodenalis* exhibited the highest resistance to ferrate with a k-value of 0.064 [L/(mg-Fe × min)] at pH = 8 and temperature = 25 °C, while human adenovirus was the most susceptible under all the testing conditions with the relatively broad k range of 5.93–36.2 [L/(mg-Fe × min)]. The treatability of the microorganisms ($\log_{10}$ inactivation) using ferrate disinfection under optimal conditions (pH = 7 and temperature = 25 °C) is plotted in Figure 1. The bacterial strains tested, including *E. coli* and *V. cholerae,* were positioned between *G. duodenalis* and human adenovirus, with *V. cholerae* showing slightly higher resistance than *E. coli* (as indicated by the round and rectangle symbols in Figure 1, respectively).

CT values corresponding to 2-, 3- and 4-log inactivations were calculated for each microorganism based on the estimated k values and $\log_{10}$ inactivations for two pH values and two temperatures (Table 2). *E. coli* and *V. cholerae* achieved more than 4-log inactivation under all testing conditions with a CT of 4.3 mg-Fe × min/L, while *G. duodenalis* cysts exhibited the highest resistance to ferrate, requiring approximately 30 times a greater CT value. For HAdV2, a maximum of over 4-log inactivation was achieved with a CT as low as 0.5 mg-Fe × min/L under optimal conditions (pH = 7 and temperature = 25 °C), whereas less than 2-log inactivation was observed at pH = 9 and temperature = 25 °C with the same CT value.

Human adenovirus is relatively susceptible to inactivation by ferrate, requiring significantly lower CT values to achieve the same level of log inactivation as other tested microorganisms. This is consistent with the findings of Schink & Waite (1980), who reported that f2 coliphage was the least resistant microorganism to ferrate compared to several species of bacteria, including *E. coli.* However, a direct comparison of the results from their study to ours regarding the rates of microbial inactivation efficacy is not feasible, as they did not use a commonly accepted model to calculate k or CT values. Our findings suggest that the resistant rank of the tested microorganisms (e.g., bacteria, viruses, and protozoan parasites) to ferrate follows a similar trend, with adenovirus being the least resistant among microorganisms tested in this study. Further study, employing a robust experimental plan, is requisite for the estimation of the k or CT values across a variety of bacteriophages. Beyond the scope of viral surrogates, the establishment of an exhaustive database documenting the inactivation potency of ferrate against a diverse range of human enteric viruses, inclusive of enteroviruses, is of paramount importance. This will serve as a pivotal reference point in the field.

Numerous studies have reported that ferrate effectively inactivates a variety of microorganisms over wide ranges of pH (Cho et al. 2006; Jiang et al. 2007; Sharma 2007; Hu et al. 2012). We found that the microbial inactivation rates increased at lower pH and higher temperature (Table 2), indicating a considerable pH and temperature dependence of many ferrate reactions. The greater inactivation at lower pH is consistent with other studies and with the premise that the ${\text{HFeO}}_{4}^{-}$ acid/base species, which dominates, relative to ${\text{FeO}}_{4}^{-}$ below pH 7.3, is a much stronger oxidant (Kazama 1994; Sharma et al. 2005). It is well established that protonated species of ferrate have a higher biocidal effect at lower pH levels (Rush et al. 1996). Although the effectiveness of ferrate disinfection is less dependent on water pH than chlorination (Jiang & Wang 2003), pH remains a crucial parameter impacting the inactivation efficacy of the tested microorganisms.

Chemical disinfectants are more effective against naked icosahedral viruses, such as human adenovirus, than gram-negative bacteria and protozoan parasites. Sharma et al. (2005) demonstrated that ferrate achieved more than 3-log inactivation of total coliforms in water. In comparison to chlorination, Jiang & Wang (2003) reported that ferrate required CT values one order of magnitude greater to achieve the same level of *E. coli* inactivation. However, ferrate demonstrated significantly greater inactivation efficacy against chlorine-resistant bacteria, such as aerobic spores and sulfite-reducing *Clostridia,* under the same testing conditions with a similar CT value of chlorine (Franklin 1998). Besides bacteria, several researchers have reported the effectiveness of ferrate in disinfecting bacteriophages f2, Qβ, and MS2 (Schink & Waite 1980; Kazama 1994, 1995; Hu et al. 2012). Ferrate was found to be slightly less effective for viral inactivation against these bacteriophages than chlorine under similar treatment conditions in buffer (Scarpino et al. 1972; Schink & Waite 1980). On the contrary, under conditions where chlorine dissipated rapidly due to reactions with natural organic matter (NOM) that is rich in municipal wastewater, ferrate required lower doses and CT (i.e., CT values) than chloramination (Cramer et al. 1976) and was as effective as ozonation (Longley et al. 1974).

While several previous studies have suggested the superior efficacy of ferrate in inactivating a variety of microorganisms, it is not feasible to compare those results with our study due to the lack of standardized reporting formats. Recently, there has been more attention paid to understanding the roles of intermediate species Fe(V) and Fe(IV), both of which have oxidative power and putative high reactivity (Sharma et al. 2022). Moreover, a recent study by Huang et al. (2018) suggested that the use of phosphate buffers at concentrations typically used in controlled lab studies, such as ours, may impart a negative effect on the oxidative capacity of a ‘ferrate-based’ process. The potential nuances of the Fe(VI)/Fe(V)/Fe(IV) interactions and how they behave in any given system further complicate direct comparisons between studies.

Ferrate possesses two advantageous properties for water treatment mechanisms: powerful oxidation and coagulation (Jiang et al. 2006). In addition to its ability to disinfect microorganisms, ferrate can remove precursors of DBPs, such as NOM, in water treatment practices, thereby mitigating DBP formation potential. Furthermore, it has been reported that ferrate does not react with bromide or NOM, indicating that it does not produce carcinogenic DBPs during the disinfection process. Due to its selective oxidation property against biomolecules, such as amino acids (Lee & von Gunten 2010), ferrate shows promise for application in the disinfection of waters that are rich in NOM. Specifically, its ability to mitigate the potential for DBP formation in the direct potable reuse of wastewater makes it an alternative disinfectant.

In contrast, ferrate also presents certain disadvantages, one of which is the formation of treatment residuals. Fe(VI) undergoes a progressive conversion to Fe(III), which may subsequently lead to the formation of sediments. Further research is warranted to devise effective strategies for the management of these treatment residuals. Furthermore, in the context of residual disinfection, the inherent instability of ferrate in aqueous solutions, attributed to its relatively rapid autodecomposition, along with the sediment formation, poses challenges to its utilization for microbial control in distribution systems as a viable alternative to chlorine. Despite the existence of research gaps, a significant proportion of research areas, including contaminant treatability, optimal treatment conditions, ferrate quantification and monitoring in water, and the evolution of its production methodologies, have been successfully demonstrated at a laboratory scale. Nevertheless, ferrate is still classified as an emerging disinfectant due to the absence of large-scale implementation (Deng & Abdel-Shafy 2024).

## 4. CONCLUSIONS

The study revealed that ferrate disinfection is effective against a variety of microorganisms, with human adenovirus being the least resistant and *G. duodenalis* exhibiting the highest resistance. In line with other studies, for the two pH values (7 and 8) and two temperatures (5 and 25 °C) examined, the inactivation rates of all tested microorganisms increased with lower pH and higher temperature. These findings suggest that ferrate disinfection could be a promising strategy for practical water treatment applications, taking into account factors such as competition with NOM and cost considerations. Nevertheless, further research is necessary to optimize this process and identify the most effective conditions for different microorganisms. Given the dual properties of ferrate, such as potent oxidation and coagulation, we also suggest further studies to evaluate its coagulation properties for enhancing treatment efficiency during water treatment processes.

## Figures and Tables

**Table 1 ∣:** Inactivation rate constant (k) [L/(mg-Fe × min)] of various microorganisms at different pH and temperatures using ferrate

	pH = 7		pH = 8		pH = 9^a^	
	5 °C	25 °C	5 °C	25 °C	5 °C	25 °C
*E. coli* O157 35150	8.64	15.7	2.95	3.02	ND	ND
*E. coli* 25922	7.21	ND	1.94	2.72	ND	ND
*E. coli* 35327	6.90	12.4	1.76	3.10	ND	ND
*V. cholerae* 6706-Smooth	3.14^b^	7.04	2.27	2.15	ND	ND
*V. cholerae* 2614-LA	ND	10.7	ND	3.05	ND	ND
*V. cholerae* 14033	ND	7.22^b^	ND	3.52	ND	ND
HAdV2	28.84	36.2	11.53	16.52	5.93	8.64
*G. duodenalis* cyst	ND	0.201^b^	ND	0.064	ND	ND

ND, not determined.

a Only human adenovirus was tested at pH = 9.

b *R*^2^ is below 0.2.

**Table 2 ∣:** CT (mg/L as Fe × min) table for inactivating various microorganisms using ferrate

	2-log (99%)				3-log (99.9%)				4-log (99.99%)			
	pH = 7		pH = 8		pH = 7		pH = 8		pH = 7		pH = 8	
	5 °C	25 °C	5 °C	25 °C	5 °C	25 °C	5 °C	25 °C	5 °C	25 °C	5 °C	25 °C
*E. coli* O157 35150	0.53	0.29	1.56	1.52	0.80	0.44	2.34	2.29	1.07	0.59	3.12	3.05
*E. coli* 25922	0.64	0.81	2.37	1.69	0.96	1.22	3.56	2.54	1.28	1.62	4.75	3.39
*E. coli* 35327	0.67	0.37	2.62	1.49	1.00	0.56	3.92	2.23	1.33	0.74	5.23	2.97
*V. cholerae* 6706-Smooth	1.47	0.65	2.03	2.14	2.20	0.98	3.04	3.21	2.93	1.31	4.06	4.28
*V. cholerae* 2614-LA	ND	0.43	ND	1.51	ND	0.65	ND	2.26	ND	0.86	ND	3.02
*V. cholerae* 14033	ND	0.64	ND	1.31	ND	0.96	ND	1.96	ND	1.28	ND	2.62
HAdV2	0.16	0.13	0.40	0.28	0.24	0.19	0.60	0.42	0.32	0.25	0.80	0.56
*G. duodenalis* cyst	ND	22.9	ND	72.0	ND	34.4	ND	107.9	ND	45.8	ND	143.9

ND, not determined.

## Data Availability

All relevant data are included in the paper or its Supplementary Information.

## HIGHLIGHTS

- Ferrate effectively inactivates a variety of tested microorganisms.
- The inactivation rates of all tested microorganisms increased with lower pH and higher temperature.
- A ferrate CT table facilitates the comparison of the effectiveness of various disinfection methods.
- Ferrate disinfection could be a promising strategy for practical water treatment applications.
